# Analysis of the Objective Internal Load in Portuguese Skydivers in the First Jump of the Day

**DOI:** 10.3390/s22093298

**Published:** 2022-04-26

**Authors:** Tiago Machado, João Serrano, Jose Pino-Ortega, Paulo Silveira, Antonio Antúnez, Sergio José Ibáñez

**Affiliations:** 1 Faculty of Sports Sciences, University of Extremadura, 10005 Cáceres, Spain; tisantosm@alumnos.unex.es; 2 Higher School of Education, Instituto Politécnico de Castelo Branco, (SHERU) Sport, Health and Exercise Research Unit, 600-266 Castelo Branco, Portugal; j.serrano@ipcb.pt (J.S.); paulo.silveira@ipcb.pt (P.S.); 3 Department of Physical Activity and Sport, University of Murcia, 30100 Murcia, Spain; josepinoortega@um.es; 4 Research Group in Optimization of Training and Sports Performance (GOERD), Faculty of Sports Sciences, University of Extremadura, 10005 Cáceres, Spain; sibanez@unex.es

**Keywords:** parachuting, objective internal load, HR, stress

## Abstract

The general objective of this study was to identify the variation in heart rate (HR) of Portuguese skydivers during 6 moments in their first jump of the day, bearing in mind the variable level of experience. Thirty-one Portuguese skydivers, 28 men and 3 women, aged between 19 and 62, participated in the study, 12 had A and B licenses (less experienced) and 19 had C and D licences (more experienced). The instrument used to record the heart rate of the skydivers at the different moments of their first jump of the day was the WIMU PRO. A repeated measures analysis of variance was used to analyse HR at different moments in the jump and its relation with the variables level of experience. Bonferroni multiple comparisons were performed to study the importance of the differences observed in HR at the different moments. The effect size was evaluated with partial eta squared. The results showed that average HR in this group of skydivers was 130 bpm, in the different moments of the jump. HR increases from the value recorded at rest until the moment of jumping from the plane and opening the parachute, reaching the highest average at that moment, then decreasing until contact with the ground. Comparing the variable, we found that the less experienced had higher HR than the more experienced at all moments during the jump. Statistically significant differences were found at the different moments of the jump, regarding HR (Max: *p* < 0.001, ƞ^2^_p_ = 0.820; Min: *p* < 0.001, ƞ^2^_p_ = 0.821; AVG: *p* < 0.001, ƞ^2^_p_ = 0.834) Level of experience with jumping moment interaction, we only verified differences related to HR Min (*p* = 0.007, ƞ^2^_p_ = 0.056),. With regard to experience, the identified differences were not statistically significant. Skydiving triggers an acute adaptive cardiovascular response which is reflected in the increase in the HR, between the moment of boarding the plane and the moment at which the parachute opens, thereafter decreasing until contact with the ground. The most experienced parachutists recorded the highest HR at the moment of landing and the least experienced at the moment of free fall.

## 1. Introduction

Skydiving is a sport with very special characteristics. The simple fact of an individual jumping out of a plane at extreme altitude, knowing that human beings do not possess the morphological characteristics for being able to fly, inevitably causes an enormous stress stimulus in the body [1]. This modality covers skydivers, tandem passengers, instructors, and skydiving students, being regulated in the Portuguese Federation of Skydiving several modalities with very particular specificities, such as: formation skydiving, freestyle skydiving, accuracy landing, speed skydiving, canopy piloting and freefly, the equipment needed is determined by the type of jump, experience of the skydiver, weather and conditions in the launch zone, it is required to use an altimeter and helmet in addition to the parachute. The parachute is constituted to the harness, main and reserve wing, it also has the AAD which is an “automatic activation device” that will automatically deploy your parachute if you or your instructor cannot deploy it manually in case of unconsciousness. The AAD monitors your altitude and rate of descent and triggers at a certain height if your descent speed is excessive, ensuring you land safely [2]. In methodological terms during the skydiving course, the position of the body is called belly flight, it is in this position that the parachutist’s first objective is to fly stably so that later he has the ability to move in all directions [2].

When thinking about the discipline of parachuting it is natural to associate it with the possibility of serious and even fatal accidents; however, many of the accidents that occur are mainly due to human error [3]. There is also evidence that over the years there has been a reduction in the number of fatal accidents, although in 2020 there were still 11 fatal accidents in the United States of America, among the millions of jumps that were made during the year [4].

In skydiving, as well as the importance of the aspects related to the flying technique, safety standards (before, during and after the jump), emergency procedures to be adopted in case of an equipment failure or human error, it is fundamental to bear in mind the management of some physiological effects, such as hypoxia which occurs at an altitude of approximately 3660 m (12,007 feet), which can result in drowsiness, and muscular and mental fatigue. Guyton and Hall, Ref. [5] report that even above 5490 m (18,011 feet) these effects can cause spasms and convulsions due to severe hypoxia (alveolar PO2 at sea level is 104 mmHg and at 6100 m it is 40 mmHg). Another important aspect is the speed of acceleration and deceleration to which the skydiver is exposed. After jumping from the plane, the speed immediately reaches 9 m per second and after 12 s of free fall and having descended 420 m, the speed reached is from 175 to 190 km/h. At the moment that the parachute opens at speeds of nearly 200 km/h the impact load on the harness is 540 kg [5].

The above-mentioned conditions show that as well as the external factors inherent in the sport, there are also internal factors that can be influenced by psychological variables [6]. Skydiving is characterised by being a discipline in which emotional management and anxiety predominate, which has an influence when trying to make assessments using heart rate (HR).

Paschoal et al. [7] reported that heart rate variability (HRV) is an evaluation tool that involves both the cardiovascular system and the autonomic nervous system (ANS), which after receiving information from the body itself and the external surroundings, responds by activating or inhibiting the two systems that comprise it, the sympathetic nervous system (SNS) and the parasympathetic nervous system (PNS). In general, these two systems intervene in a coordinated manner so that the response is adequate for different situations [8]. Skydiving, as an extreme challenge, triggers the activation of the SNS, specifically in the increase of the HR, thus facilitating both physical and psychological changes in order to endow the individual with the capacity for flight or fight, as an adaptation strategy for challenging situations [9,10]. One of the most distinctive characteristics of the ANS is the speed and intensity with which it can change HR, in a matter of 3 to 5 s increasing it to double the normal rate, as well as reducing it in 4 to 5 s enough to even cause fainting. Strong vagal stimulation (parasympathetic) releases acetylcholine in the vagal nerve endings, decreasing HR and the excitability of the A-V node, which slows down the transmission of the heart impulse to the ventricles. In general, the PNS acts in opposition to the SNS, inverting the generated response, decreasing HR and arterial pressure and sweating [11].

Measurement of HR has been widely used to study metabolism in sports practice regarding the intensity of the physical effort or as an indirect evaluation of the energy output during the activity [12]. However, there are several factors such as age, sex, motivation/anxiety, medication, caffeine and tobacco or the environmental conditions that can influence the result. Alves [13] states that, with ageing, maximum HR decreases and women have a higher HR.

Tintoré et al. [14] carried out one of the first studies in which they proposed to analyse, using an electrocardiogram, the variations in HR that occur during skydiving, that is, during the moment of free fall, assessing the effect of emotional stress on the release of catecholamines in a discipline where physical effort is low. Ref. [15] also analysed the changes in heart rate among novices and experienced parachutists from the moment when they arrived at the hangar until the moment at which they jumped. The investigations show that one of the responses to the steady state during the jump is an increase in HR [16], but it would be difficult to analyse and control all the variables simultaneously (temperature, altitude, personality characteristics, BMI) which can trigger physiological changes at the cardiovascular level; however it is very pertinent to analyse in greater detail HR variations at different moments.

Similar to the studies of Mazurek et al. [17] and Cavalade et al. [18] who divided a parachute jump into different moments (time line with measurements) for a more detailed analysis, the present study also used this procedure to analyse HR before, during and after a parachute jump.

In spite of the scientific contributions of the last century, Machado et al. [18,19] found that the research carried out since 2000 on parachuting, from a physiological point of view, that is HR, was still scarce. From the searches carried out, no investigations focused on the objective internal load of Portuguese parachutists were found. The general objective of this study was to identify the variation in the HR of Portuguese skydivers during the 6 moments of the first jump of the day, bearing in mind the variable level of experience. Starting from the central aim, four specific objectives were formulated:; (i) to describe the HR values of the Portuguese skydivers during 6 moments of the first jump of the day, bearing in mind the variables of level of experience; (ii) to identify the differences in the HR values of the Portuguese skydivers during 6 moments of the first jump of the day, bearing in mind the variables of level of experience; and (iii) to analyse the interaction among HR, the moment of the jump and the variable level of experience.

## 2. Materials and Methods

### 2.1. Design

It is a descriptive study in order to record the HR of the skydivers at different moments of the jump, having sex and experience as variables. No intervention was performed during the study, so it was given a ecological treatment [20].

### 2.2. Characterisation of the Sample

All the skydivers who met the inclusion criteria (necessary requirement would be not to have a time period greater than 1 month without having jumped) who were at the Moitas skydiving center in Proença-a-Nova participated in the study. Thirty-one Portuguese skydivers between the ages of 19 and 62 (*M* = 42.1; *SD* = 12.34) participated in the study, 28 (90%) were men with an average BMI of 25.07, and 3 (10%) were women with an average BMI of 22.40. The level of experience was determined according to the federative levels of classification: all those with A and B licences (12 athletes), with an average of 88 jumps and 1 year and 3 months of practice in the modalitywere considered less experienced, and those with C and D licences (19 athletes with an average of 1785 jumps and 19 year and 7 months of practice in the modality, were considered more experienced. According to the Contemporary Portugal database in the year 2020, 603 athletes were registered with the Portuguese Parachuting Federation [21]. 

### 2.3. Instruments

The instrument used to collect the data was the WIMU PRO which is composed of four 3D accelerometers, and other sensors such as gyroscopes, magnetometers, barometers, GPS and UWB, that detect and measure movement using a microelectromechanical system with an adjustable sampling frequency of 10 to 1000 Hz. Each device has its own GHz microprocessor, flash memory and high velocity USB interface to record, store and upload data. The device has an internal battery with 4 h of autonomy, weighs 70gr and measures 81 mm × 45 mm × 16 mm [22]. A GARMIN heart rate band was used to record HR and data were stored in the WIMU Pro device as both instruments are connected. The WIMUO Pro device is a valid and reliable instrument for assessing flight time, being a useful tool with the enormous advantage of not needing cables, which permits freedom of movement [23] but, above all, avoiding compromising the safety of the skydivers as there are no cables which could become entangled in their equipment. 

A sociodemographic questionnaire was used to classify the skydivers (sex, nationality, number of sports licence and experience in the sports activity—level of federative qualification, number of jumps, date of last jump and year of starting the activity). 

### 2.4. Measurements Variables

The dependent variable in this study was heart rate, recorded as: maximum heart rate (HR max), Minimum heart rate (HR min) and Average heart rate (HR AVG). 

Two independent variables were used: level of experience and moment of the jump.

Level of experience. The level of experience was classified according to the federative licence: (i) Less experienced: all the skydivers who were in the training phase and had an A and B licence; and (ii) More experienced: all the skydivers who had a C and D licence.

Moment of the jump. Six moments in the jump were defined, following Cavalade et al. [16]:

Moment 0: Fifteen minutes before boarding the plane, the subjects sit resting for two minutes.
Moment 1: The take-off phase.
Moment 2: Two minutes before the skydivers jump from the plane (when it is at maximum altitude).
Moment 3: From leaving the plane until the parachute opens (free fall phase). This phase lasted an average of 50 to 60 s, depending on the free fall velocity and the time the parachute took to open.
Moment 4: Two minutes after the deployment of the parachute (canopy flying).
Moment 5: Two minutes after contact with the ground.

### 2.5. Procedures

The first step was to establish contact with the President of the Portuguese Skydiving Federation (FPP), conveying to him what was intended with the study (objectives, procedures and potential). Subsequently, the Skydiving School—SkyFunCenter, which carries out its activities at the Moitas aerodrome (Proença-a-Nova) was contacted in order to authorize the collection of data on its premises, after a detailed explanation of the study we intended to carry out. On the day of data collection, all participants were duly informed of all the details of the study they were going to be part of. All participants were also informed that they would perform the belly flight individually or in groups of 2 with a departure interval of 7 s to ensure complete safety. After this phase of information and clarification, the skydivers who participated in the study answered the sociodemographic questionnaire and performed the body assessment. Finally, WIMU devices and heart rate monitor bands were placed on the skydivers. All followed the same protocol for data collection, from resting 15 min before the jump, to measurement 2 min after contact with the ground.

At the end of the first jump, approximately 30 min after landing, the skydivers in a group with the investigators shared relevant information from all moments of the jump (inside the aircraft, exit moment, during the free fall, at the opening of the parachute, during canopy flight and landing). The ground wind speed was 3 m/s and the temperature was 13 °C (data from the Moitas Aerodrome meteorological center), at the time of the jump. The aircraft used was the Cessna 208 Caravan I and the parachute drop altitude was 3962 mt (13,000 ft). All skydivers deployments were performed at a safe altitude (between 5000 feet to 3500 feet) at ground level.

The study was authorized by the Bioethics and Biosafety Commission of the University of Extremadura (Registration Number: 205/2020).

### 2.6. Data Analysis

Different hypothesis tests were used to define the appropriate models to be used: the Shapiro-Wilk test and Leven’s test to prove homogeneity of the variances [24], as well as basic methods for exploratory and descriptive analysis. A repeated measures analysis of variance was used to analyse HR at the different moments of the jump and its relation with the variable level of experience, together with the Mauchly’s sphericity test (HR min) and Greenhouse-Geisser epsilon (HR max and HR AVG). The Bonferroni correction was also performed to study the significance of the differences observed in HR at the different moments. Effect size was assessed using partial eta squared [24].

The Levene test confirmed the assumption of homogeneity of variances at all the moments and in all the HR measures (Max. Min. AVG). 

The Mauchly test of sphericity showed that the assumption of sphericity was only verified with regard to HR min (*p* = 0.081) and was not evident for the variables of HR Max (*p* = 0.001) and HR AVG (*p* = 0.002). Data were processed using SPSS v 25.0 (IBM Corp., 2017. IBM SPSS Statistics para Windows, version 25.0, IBM Corp, Armonk, NY, USA).

## 3. Results

Table 1 shows that HR AVG for the whole sample was 130 bpm, HR Max 142 bpm and HR Min of 120. Regarding the level of experience, the results of HR max, min and AVG were quite similar in both groups.

With regard to the level of experience (Table 2), it was found that the HR AVG of the most experienced skydivers recorded a considerable increase (from 95 bpm to 150 bpm) between moments 0 and 4, decreasing in moments 4 to 5 (from 150 bpm to 137 bpm) Regarding the less experienced, HR AVG also recorded a considerable increase from moments 0 to 3 (from 102 bpm to 157 bpm) and decreased between moments 3 and 5. Regarding the different moments, the tendency was identical to that related to the sex variable, however, between moments 3 and 4, the more experienced group showed stability in their HR, The standard deviation (SD) shows that the most experienced group was the most homogeneous, with regard to HR Max.

**Figure 1:**
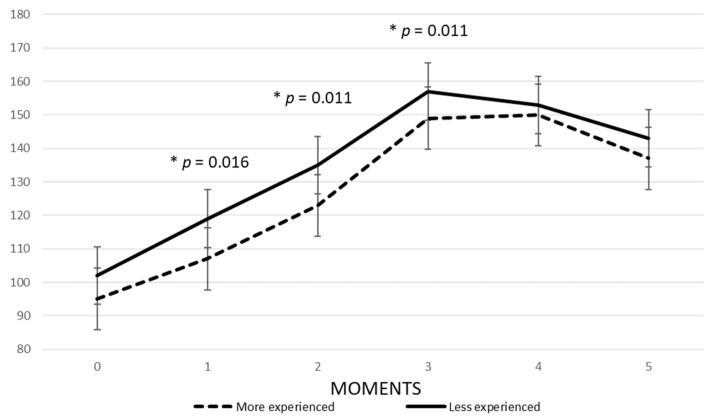
HR AVG at different moments of the jump related to the variable level of experience. Moment 0: Fifteen minutes before boarding; Moment 1: The take-off phase; Moment 2: Two minutes before the skydivers jump from the plane; Moment 3: free fall phase; Moment 4: Two minutes after the deployment of the parachute (canopy flying); Moment 5: Two minutes after contact with the ground. Time points 1, 2 and 3 show the statistically significant differences (*).

Figure 1 shows that at all the moments of the jump the more experienced skydivers recorded a lower HR compared to the less experienced. In addition, statistically significant differences were identified between the two groups of skydivers at moment 1, the take-off phase (t = 2.264; *p* = 0.016); moment 2, two minutes before the skydivers jump from the plane (t = 2.586; *p* = 0.011); and moment 3, the free fall phase (t = 2.631; *p* = 0.011). An interesting aspect is that at moment 4 the HR of both groups became more similar.

Therefore, assuming sphericity for HR Min and using the Greenhouse-Geisser epsilon for HR Max and AVG (Table 3), we conclude that the variable moment of the jump produced a significant effect on HR (Max: *p* < 0.001, ƞ^2^_p_ = 0.820; Min: *p* < 0.001, ƞ^2^_p_ = 0.821; AVG: *p* < 0.001, ƞ^2^_p_ = 0.834), of interaction between the moments of the jump and level of experience (Max: *p* = 0.681, ƞ^2^_p_ = 0.008; Min: *p* = 0.007, ƞ^2^_p_ = 0.056; AVG: *p* = 0.189, ƞ^2^_p_ = 0.028), we found that the jump moment and level of experience has a significant effect on the HR Min.

Through the multiple Bonferroni comparisons, it can be seen that for each of the HR variables (Min, Max, AVG) there are several pairs of moments in the jump in which the differences are statistically very significant. Analysing HR AVG, significant differences only failed to be found in moments 2 and 5 (*p* = 0.221); and 3 and 4 (*p* > 1.0). The same tendency is true for HR Min, at the same moments (*p* > 1.0). With respect to HR Max, moments 3 and 4, 3 and 5, 4 and 5 (*p* > 1.0) and moments 2 and 5 (*p* = 0.081) showed no statistically different differences. These results reinforce the finding that HR between moments 3 and 4 is quite similar, with no differences between the moments at which the skydivers are in free fall and the two subsequent minutes after the opening of the parachute when they are in the canopy flight phase. The remaining pairs of moments showed very significant differences (*p* < 0.01), with the exception of the HR Max variable between moments 2 and 4 (*p* = 0.014), HR Min between moments 0 and 1 in HR Min (*p* = 0.022) and HR AVG between moments 3 and 5 (*p* = 0.012) and 4 and 5 (*p* = 0.019) which recorded significant differences (*p* < 0.05). That is to say that from the boarding of the plane until the moment of free fall and the opening of the parachute, HR (Max, Min, AVG) increases, showing statistically significant differences. From that moment on, HR increases less or even decreases, but not significantly. 

## 4. Discussion

The data suggest that a parachute jump provokes a considerable increase in the internal load from moment that the plane takes off until the moment of free fall, stabilising immediately after the opening of the parachute and decreasing after contact with the ground (landing). 

HR recorded at the different moments reveals a tendency to increase, from moments 0 to 3 (before boarding the plane until the moment the parachute opens) and a slight decrease between moments 3 to 5 (time of the opening of the parachute and contact with the ground). It is important to remark that moment 3 corresponds to the free fall and opening of the parachute, which means that this stage is when the skydivers reach speeds of over 200 km/h, and open the parachute, a moment of extreme tension, and maximum levels of concentration. An interesting common result among the men and women is related to moment 4 which corresponds to the two minutes following the opening of the parachute, in which the HR AVG values remain practically identical to those of the previous moment.

The results show a significant increase in HR between moments 0 and 1 (measurement at rest and take-off of the plane) and 2–3 (inside the plane at maximum altitude and moment at which the parachute opens). The large initial increase in HR can be explained by the fact that the take-off of the plane is considered one of the most critical moments that generate most airplane accidents [25]. All the skydivers in their training are taught to deal with emergency situations and the premature departure of the plane due to breakdown is one of the very focused points, and that only from 1500 ft can you leave the plane immediately activating the reserve parachute you have a much shorter opening time [2]. On the other hand, this increase in HR in the initial phase could also be explained by the procedures learned by the skydivers in training, which says that if there is a situation of mechanical failure in the plane, only if they are above an altitude of 1500 feet can they make an emergency exit from the plane directly opening the reserve parachute. Moment 2 begins when the pilot informs them that there are two minutes left before the door of the plane will open and the skydivers can jump out (the phase of the highest altitude), which triggers physiological changes as it is the moment when the skydivers make the final check of their equipment and mentally prepare to adopt emergency procedures if the need arises González-Moro, et al. [26] also recorded the most significant increase between the moment before jumping from the plane and the moment of free fall. Analysing HR Max in moments 3 and 4, we find that, according to Maglischo [27] the HR values in moments 3 and 4 are very near to the anaerobic threshold. Ref. [28] concluded that the group of skydivers in comparison to athletes from other disciplines, recorded a significant increase in sympathetic cardiovascular control and a much lower vagal influence, thus generating a lower HRV and increase in HR not only during free fall, but also in preparation for the jump. The results show that parachuting is a discipline that triggers changes in HR level, that is, its significant increase, especially from the moment of boarding the plane until exiting it (during this period the skydiver is seated in the plane), which could indicate that skydiving generates greater stress provoking constant adaptive physiological responses. With regard to HR AVG there is a slight difference in the variable level of experience, with the more experienced recording a lower HR. This result contradicts Allison et al. [10] who obtained higher HR values in more experienced skydivers before, during and after a jump. Although there is a small difference in relation to the level of experience, it is not a variable that produces statistically significant differences regarding HR, which suggests that repeated exposure to stressing situations is not a factor that models HR reactivity. 

Leach and Griffith [29] in their study, found that the HR of more and less experienced parachutists was high, which may suggest that the activation mechanism of the SNS with the increase of HR may not only be due to anxiety or stress. Meyer et al. [30] concluded that the greater experience of the parachutists alters emotional and physiological stimulation, but does not totally extinguish the reactivity of cortisol. The release of epinephrine and norepinephrine also known as adrenaline and noradrenaline, are physiological reactions generated in situations of stress [31] and in the control of anxiety and attention [32], possibly the most common response in less experienced parachutists. But the release of these neurotransmitters in experienced parachutists may be associated with the ANS, as a modulation of reward in the brain, dopamine is a hormone that is a precursor of adrenaline, which can generate sudden and intense sensations of joy [33]. Jones et al. [34] add that there exists a complex relation among the characteristics of the personality, the need for physiological activation and performance evolution in response to motivations and needs regarding the practice of extreme sports. Physiological activation called by some authors “adrenaline rushes”, in which the practice of these disciplines becomes a desire or need for many individuals [35], can also be a reason for greater or lesser sympathetic modulation in the PNS. A study by Cavalade et al. [18] with 18 experienced skydivers (with more than 300 jumps) recorded a HR AVG value of 104 bpm, that is 30 bpm less than the group of experienced skydivers in the present study. The investigations by Hynynen et al. [36] and Mazurek et al. [17] also found a higher HR in the less experienced group, even showing statistically significant differences among them. The results of the study indicate that the level of experience, which is inherently related with the number of jumps carried out, is a variable which interferes with the HR; however, we assume that the difference between both groups was not greater due to the fact that the less experienced had already made several jumps, so that they had some experience of the discipline.

The results obtained in relation to the HR at the different moments of the jump reinforce the theory that parachuting provokes in its practitioners a gradual increase in HR, from the moment of preparation/checking of equipment, boarding the plane, jumping from the plane and the moment of free fall, with the values stabilising after the opening of the parachute until the landing, only decreasing from then on [10,14,37]. A possible explanation shown by [38] is related to the increase of hypoxia caused by the rise in altitude during the flight which provokes a sudden acceleration of heart rate. Clemente-Suárez, et al. [39] also analysed the HR variable, recording much lower values both before and after the jump, which did not surpass 100 bpm but with the particularity that the jumps were made with automatic opening of the parachute and at a low altitude (500 m). This demonstrates that the physiological theory of the decrease in O2 with altitude is valid but does not explain the reasons why in the present study between 0 and 1 (with the skydivers still on the ground) HR AVG recorded an increase of 17 bpm in the less experienced and 12 bpm in the more experienced. The different moments of the jump are differentiated by their specificity regarding the tasks or procedures to be carried out, while at moment 0 the focus is on checking and adjusting the equipment, for example, at moment 4 attention is paid to the correct verification of the opening of the parachute. However, we can see that at moment 3 (free fall) HR AVG is greater in less experienced, with the exception of the more experienced, who record the highest HR AVG at moment 4. 

One of the possible explanations is also due to the laws of physics, because, especially in more experienced skydivers, because the size of the wing of the main parachute is smaller the drag is lower which gives rise to a much higher velocity of descent. Another possible explanation is the fact that it is the moment before the landing and generates high levels of stress, as there is a need to execute the landing circuit perfectly to make contact with the ground with total safety (González-Moro et al. 2020 [25]). Taelman et al. [40] reported changes in HR and HRV in a group of resting subjects who were subjected to a stressing mental test, which suggests that carrying out mental tasks triggers physiological oscillations, so HR and HRV have the potential to measure stress levels This can be seen as the body’s response to the high secretions of adrenaline and noradrenaline, which add together as a result of the stress factor because any error in the manoeuvre could prove fatal [41]. This factor is reinforced by several investigations that analyse the moments at which the most serious accidents occur, and it is unanimously agreed that the highest percentages are at the moment of landing, followed by the moment the parachute opens (anomalies in the opening, collisions, among other situations) and hitting the plane while jumping out [42,43].

Analysing the different moments of the jump, we observed that statistically significant differences do not exist only between moments 3 and 4 regarding HR (Max., Min and AVG), which correspond to the moment of free fall and the two minutes following the opening of the parachute. A possible explanation is that the two minutes following the opening of the parachute are characterised by two unique aspects compared to the other moments: (i) the skydivers in this phase are above the earth and outside the plane (free fall and canopy flight); (ii) there is great demand regarding concentration and decision making, specifically in the opening of the parachute and the flight of the sail before landing. Mazurek et al. [17] also recorded significant differences among the different moments of the jump. Hynynen et al. [36] reported statistically significant differences of HR among a group of experienced and novice parachutists, with greater relevance when jumping from the plane and landing. 

For future research it would be relevant to study the relation among HR at different moments in different jumps on a typical parachuting day. Analysing individually the different disciplines (precision, free flight, flight training, speed) of parachuting also at the different moments would be another excellent contribution; as would studying other variables related to personality, characteristics and states of anxiety of the practitioner. 

## 5. Conclusions

Based on the results of this research, the HR AVG of the skydivers who participated in this study increased significantly from their boarding the plane until the moment of free fall, stabilising between this moment and after the opening of the parachute, to decrease slightly after the landing. 

HR AVG of the less experienced skydivers was higher at all times during the jump than in the more experienced. Statistically significant differences were recorded in relation to HR at different moments of the jump.

We did not identify in the HR (Max, AVG) significant effects caused by the level of experience or the interaction between moment of jump, but in HR Min there were significant effects.

Skydiving triggers an acute adaptive cardiovascular response that is reflected in the increase in HR, between the moment of boarding the plan and the moment when the parachute opens, thereafter decreasing until contact with the ground. 

Finally, the main limitation of the study was the small sample of women, but this was to be expected due to the lower participation of women in this discipline. Another limitation was the difficulty to analyse HR in a period of 24 h preceding a day of skydiving.

## Figures and Tables

**Table 1:** HR (Max., Min. AVG) of the whole sample and according to level of experience.

			Level of Experience			
	Total Sample		More Experienced		Less Experienced	
**HR**	*M*	*SD*	*M*	*SD*	*M*	*SD*
**Max**	142	4.68	138	5.86	140	3.78
**Min**	120	4.6	114	5.75	118	3.71
**AVG**	130	4.51	125	5.64	128	3.64

**Table 2:** HR at the moments of a jump in relation to level of experience.

	More Experienced											
	0		1		2		3		4		5	
	*M*	*SD*	*M*	*SD*	*M*	*SD*	*M*	*SD*	*M*	*SD*	*M*	*SD*
**Max**	92	14.5	118	14.6	139	8.92	158	9.79	162	8.31	157	9.98
**Min**	88	16	95	17	112	12.8	137	12.4	141	9.93	122	11.4
**AVG**	95	16.5	107	15.2	123	11.5	149	10	150	9.28	137	10.9
	**Less Experienced**											
	**0**		**1**		**2**		**3**		**4**		**5**	
	*M*	*SD*	*M*	*SD*	*M*	*SD*	*M*	*SD*	*M*	*SD*	*M*	*SD*
**Max**	103	19.1	128	16.3	146	15.1	159	11.9	159	17.2	155	17.8
**Min**	96	16.8	110	13.5	126	16	146	17.2	143	17.9	130	13
**AVG**	102	16.5	119	13.8	135	14.7	157	14.3	153	16.2	143	12.4

**Table 3:** Level of significance of the variables with HR.

Variable	HR	Measure	df	F	Sig.	Partial Eta Squared	Power Observed
Moment	Max	Greenhouse-Geisser	2.534	255.904	0.000 *	0.820	1.000
	Min	Sphericity ass.	5	257.554	0.000 *	0.821	1.000
	AVG	Greenhouse-Geisser	2.814	282.332	0.000 *	0.834	1.000
Moment * experience	Max	Greenhouse-Geisser	2.534	0.457	0.681	0.008	0.133
	Min	Sphericity ass.	5	3.291	0.007	0.056	0.893
	AVG	Greenhouse-Geisser	2.814	1.621	0.189	0.028	0.406

Using B* * Statistical differences (*p* < 0.001).

## Data Availability

Not applicable.
